# Excess deaths among adults in the state of Santa Catarina: an ecological study during the COVID-19 pandemic, Brazil, 2020-2021

**DOI:** 10.1590/S2237-96222023000200003

**Published:** 2023-07-14

**Authors:** Rebeca Heyse Holzbach, Gabriel Resun Gomes da Silva, Jean Carlos Bianchi, Danúbia Hillesheim, Fabrício Augusto Menegon, Ana Luiza de Lima Curi Hallal

**Affiliations:** 1 Universidade Federal de Santa Catarina, Curso de Medicina, Florianópolis, SC, Brazil; 2 Universidade Federal de Santa Catarina, Programa de Pós-Graduação em Saúde Coletiva, Florianópolis, SC, Brazil; 3 Universidade Federal de Santa Catarina, Departamento de Saúde Pública, Florianópolis, SC, Brazil

**Keywords:** COVID-19, Coronavirus, Excess Mortality, Mortality, Descriptive Epidemiology, COVID-19, Coronavirus, Exceso de Mortalidad, Mortalidad, Epidemiología Descriptiva, Covid-19, Coronavírus, Excesso de Mortalidade, Mortalidade, Epidemiologia Descritiva

## Abstract

**Objective:**

to estimate excess deaths during the COVID-19 pandemic in the state of Santa Catarina and its macro-regions, Brazil, 2020-2021.

**Methods:**

this was an ecological study, using data from the Mortality Information System; excess deaths in adults were calculated by the difference between the observed number of deaths and expected number of deaths, taking into account the average of deaths that occurred between 2015 and 2019; the variables “macro-region of residence”, “quarter”, “month”, “sex” and “age group” were analyzed; data were analyzed in a descriptive manner.

**Results:**

a total of 6,315 excess deaths in 2020 and 17,391 in 2021, mostly in males (57.4%) and those aged 60 years and older (74.0%); macro-regions and periods with the greatest excess deaths were those in which there were most deaths due to COVID-19; the greatest excess deaths occurred in March 2021 (n = 4,207), with a progressive decrease until the end of the year.

**Conclusion:**

there were excess deaths in the state of Santa Catarina and in all its macro-regions during the COVID-19 pandemic.

## RESUMO

**Objetivo:**

estimar o excesso de óbitos durante a pandemia de covid-19 em Santa Catarina e suas macrorregiões, Brasil, 2020-2021.

**Métodos:**

estudo ecológico, com dados do Sistema de Informações sobre Mortalidade; o excesso de óbitos em adultos foi calculado pela diferença entre óbitos observados e óbitos esperados, considerando-se a média das mortes ocorridas entre 2015 e 2019; foram analisadas as variáveis “macrorregião de residência”, “trimestre”, “mês”, “sexo” e “faixa etária”; os dados foram analisados descritivamente.

**Resultados:**

excesso de 6.315 óbitos em 2020 e de 17.391 em 2021, majoritariamente no sexo masculino (57,4%) e nas idades acima de 60 anos (74,0%); as macrorregiões e períodos com maior excedente foram aqueles com mais mortes por covid-19; o maior excesso ocorreu em março de 2021 (n = 4.207), com queda progressiva até o final do ano.

**Conclusão:**houve excesso de óbitos em Santa Catarina e todas as suas macrorregiões durante a pandemia de covid-19.

## RESUMEN

**Objetivo:**

estimar el exceso de muertes en Santa Catarina y macrorregiones durante la pandemia de COVID-19, en los años 2020 y 2021.

**Métodos:**

estudio ecológico utilizando Sistema de Información de Mortalidad. El exceso de defunciones en adultos se calculó por la diferencia entre defunciones observadas y esperadas, considerando el promedio de defunciones entre 2015 y 2019. Las variables analizadas fueron: macrorregión de residencia, trimestre, mes, sexo y grupo de edad. Los datos se analizaron descriptivamente.

**Resultados:**

el exceso fue de 6.315 defunciones en 2020 y 17.391 en 2021, mayor en varones (57,4%) y mayores de 60 años (74,0%). Las macrorregiones y periodos con mayor superávit fueron los que registraron más muertes por COVID-19. El mayor exceso de muertes ocurrió en marzo de 2021 (n=4.207), con una disminución progresiva hasta el final del año.

**Conclusión:**

hubo exceso de muertes en Santa Catarina y en todas macrorregiones durante la pandemia de COVID-19.

**Table:** 

Study contributions	
**Main results**	The number of deaths in the state of Santa Catarina was higher than expected in 2020 (16.3%) and 2021 (45.1%), when compared to the pre-pandemic years. There were excess deaths in all macro-regions of the state, and it was more significant where the highest number of COVID-19 deaths occurred.
**Implications for services**	The excess deaths found reflect the underreporting of COVID-19 cases, in addition to lack of health care for people with other diseases. The services should expand their capacity for health care, testing, and continuity of care.
**Perspectives**	Organization of services with an emphasis on case notifications, access and capacity of active search for health care for individuals in social isolation. Future studies should evaluate excess deaths in Santa Catarina taking into consideration specific causes.

## INTRODUCTION

In 2021, Brazil became the epicenter of the COVID-19 pandemic, reaching an average of 4,000 deaths in 24 hours.^1^ By the end of June 2022, Brazil recorded 669,530 deaths from COVID-19.^2^ However, the data seem to underestimate the impact of COVID-19 on the country, not only because of significant underreporting, but also for the indirect consequences of the pandemic.^3^ ^,^ ^4^

Problems related to resource scarcity, conflicts of interest involving political parties, lack of coordination between the spheres of the executive branch and non-compliance with social isolation measures culminated in what is considered the greatest healthcare and hospital collapse in Brazil’s history.^3^ ^,^ ^5^ ^,^ ^6^ Overloaded health system, delay in receiving care and difficulties in accessing services probably led to greater morbidity and mortality from other diseases, as observed in countries such as the United States and England.^3^ ^,^ ^7^ ^-^ ^9^

With regard to the Brazilian scenario, disorganization of services, lack of coordination among federal entities and excessive distances to be traveled when seeking care were other factors that may have contributed to an increase in morbidity and mortality.^6^ ^,^ ^10^ Especially among people with chronic diseases and groups at risk from COVID-19, there was a low demand for health services as a result of more severe measures of social isolation.^3^ Furthermore, it is noteworthy that the supply of essential services for other conditions and injuries has been reduced, and even interrupted in several regions,^4^ impairing the continuity of care and increasing the risk of death from other causes.^3^ ^,^ ^8^

Estimating the effects of the pandemic has therefore become a challenge. Studies that take into account only the notifications of deaths due to COVID-19, and disregard the indirect consequences generated by the disease, underestimate the impact of the pandemic on mortality.^11^ ^,^ ^12^ Thus, the World Health Organization (WHO) recommends the calculation of excess deaths as a powerful tool to estimate the real impact of the pandemic on overall mortality among the population.^11^ ^-^ ^13^

The objective of this study was to estimate excess deaths in the state of Santa Catarina and its macro-regions during the COVID-19 pandemic between 2020 and 2021.

## METHODS

This was an ecological study conducted with the death notifications registered on the Mortality Information System (Sistema de Informações sobre Mortalidade - SIM) between January 1, 2020 and December 31, 2021, with the state of Santa Catarina as a unit of analysis. Data were accessed on January 28, 2022, via the Brazilian National Health System Information Technology Department (Departamento de Informática do Sistema Único de Saúde - DATASUS) website, (https://datasus.saude.gov.br/).

The following variables were analyzed:

- sex (male; female);
- Macro-region of residence in the state of Santa Catarina (Great West; Midwest, and Serra; Foz do Rio Itajaí; Vale do Itajaí ; Greater Florianópolis; South; Northeast and North Plateau);
- age group (in years: 20 to 29; 30 to 39; 40 to 49; 50 to 59; 60 to 69; 70 to 79; 80 and older);
- quarter (1^st^; 2^nd^; 3^rd^; 4^th^); and
- calendar months.

Individuals from 0 to 19 years of age were excluded from the study, since COVID-19 manifests itself differently in children and adolescents,^14^ while adults aged 20 years or older and whose death was recorded during the study period were included.

The expected number of deaths was calculated by the simple average of the number of deaths in the years 2015 and 2019, that is, the number of deaths in each of the five years was added (according to the location and period determined) and the result of the addition was divided by 5, according to the methodology proposed by other authors.^12^ ^,^ ^15^ Excess deaths were estimated by the difference between the observed number of deaths in 2020 and 2021 and expected number of deaths for the same period. The mathematical ratio between the observed number of deaths and expected number of deaths in the period was calculated for each quarter of 2020 and 2021, according to the macro-region.

Data analysis was performed using Microsoft Office Excel 2016. Absolute frequencies (n), averages, standard deviation (SD) and the percentage change of the data was calculated. The percentage change of excess deaths between 2020 and 2021, in each macro-region, was calculated by using the following formula:

Percentage change = (Excess deaths in 2021 - Excess deaths in 2020) x 100÷ Excess deaths in 2020

This study was not submitted for approval of a Research Ethics Committee, given that information from secondary databases, without identification of individuals, and in the public domain was used.

## RESULTS

In the five years prior to the COVID-19 pandemic, the average number of deaths in the state of Santa Catarina was 38,522 [(SD) = 1,546.3]. In 2020, there was a 16.3% increase in the number of deaths in relation to this average; and in 2021, a 45.1% increase. In all macro-regions the number of deaths was higher than expected (Table 1).

As for the year 2020, there were 6,315 excess deaths. The South (1,362) and Greater Florianópolis (1,146) macro-regions showed the largest increase, in addition to the highest number of notifications of deaths due to COVID-19. In 2021, there were 17,391 excess deaths, and this number was more significant in the Northeast and North Plateau (3,515), South (2,855) and Midwest and Serra (2,691) macro-regions, as well as there were more deaths due to COVID-19. It could be seen an increase in death ratios in most macro-regions, in almost all quarters; with the exception of the 2^nd^ quarter of 2020 (Tables 1 and 2).

**Figure 1:**
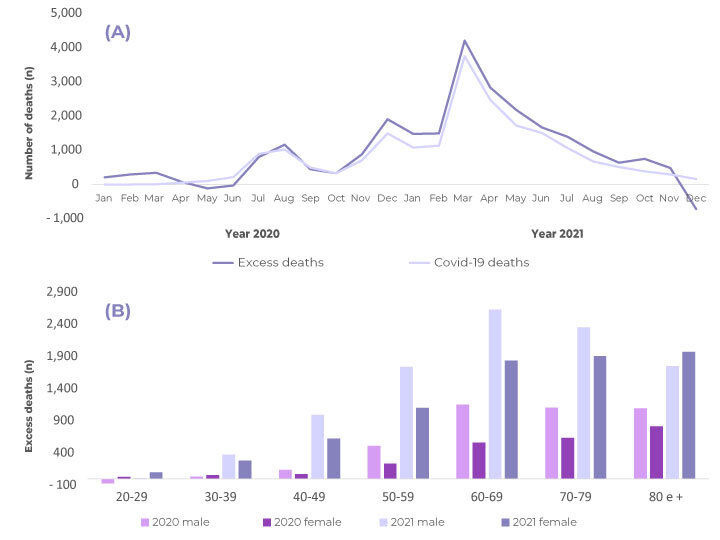
- Evolution of COVID-19 deaths and excess deaths in the period (a) and distribution of excess deaths according to sex and age group (b), state of Santa Catarina, Brazil, 2020-2021

The highest number of excess deaths was observed in the periods when there were more deaths due to COVID-19. In 2020, the highest excess deaths occurred during two peaks, one in July-August and another at the end of the year. The highest values for both excess deaths (4,207) and deaths due to COVID-19 (3,752), occurred in March 2021 (Figure 1a).

Excess deaths were found for all age groups, both in 2020 and 2021; with the exception of the male age group 20-29 in 2020. Excess deaths were higher in males (57.4%), in all other age groups, in both years, when compared to excess deaths in females. For both sexes, it could be seen excess deaths in the population aged 60 years and older (74.0%) (Figure 1b).

**Table 1:** - Overall deaths and COVID-19 deaths reported by macro-region of residence and year, state of Santa Catarina, Brazil, 2015-2021

Macro-region of residence	2015	2016	2017	2018	2019	Average^a^ (SD^b^)	2020		2021	
							Overall	Covid-19	Overall	Covid-19
Great West	4,103	4,473	4,309	4,353	4,350	4,317 (134.7)	4,666	400	6,613	1,904
Midwet and Serra	5,482	5,723	5,690	5,818	5,989	5,740 (185.4)	6,504	596	8,431	2,154
Foz do Rio Itajaí	3,137	3,400	3,414	3,511	3,621	3,416 (179.7)	4,331	737	5,254	1,461
Vale do Itajaí	5,564	5,877	5,716	5,968	5,947	5,814 (171.4)	6,545	698	7,755	1,572
Great Florianópolis	5,563	5,947	6,106	6,033	6,250	5,979 (258.1)	7,125	852	8,238	1,876
South	5,478	5,943	5,745	6,141	6,306	5,922 (325.7)	7,284	1,088	8,777	2,227
Northeast and North Plateau	6,839	7,215	7,182	7,612	7,804	7,330 (380.9)	8,382	909	10,845	3,347
TOTAL	36,166	38,578	38,162	39,436	40,267	38,522 (1,546.3)	44,837	5,280	55,913	14,541

(a) Average of years in the period 2015-2019; SD = standard deviation. Source: Mortality Information System (Sistema de Informações sobre Mortalidade - SIM).

**Table 2:** - The ratio between the observed number of deaths and expected number of deaths by quarter, excess deaths and percentage change according to macro-region, state of Santa Catarina, Brazil, 2020-2021

Macro-region	1^st^ quarter		2^nd^ quarter		3^rd^ quarter		4^th^ quarter		Excess deaths		
	2020	2021	2020	2021	2020	2021	2020	2021	2020 (n)	2021 (n)	Percentage change^a^ (%)
Great west	1.08	2.12	0.96	1.60	1.08	1.28	1.20	1.19	349	2,296	557.9
Midwest and Serra	1.13	1.71	1.01	1.84	1.12	1.26	1.28	1.08	764	2,691	252.2
Foz do Rio Itajaí	1.10	1.84	1.16	1.70	1.37	1.38	1.42	1.25	915	1,838	100.9
Vale do Itajaí	1.04	1.56	0.93	1.46	1.20	1.26	1.33	1.07	731	1,941	165.5
Great Florianópolis	1.08	1.97	1.02	1.41	1.28	1.21	1.37	0.97	1,146	2,259	97.1
South	1.12	1.76	0.95	1.80	1.34	1.23	1.50	1.17	1,362	2,855	109.6
Northeast e North Plateau	1.11	1.84	0.99	1.89	1.24	1.39	1.23	0.83	1,052	3,515	234.2
TOTAL	1.10	1.82	0.99	1.68	1.23	1.29	1.33	1.06	6,315	17,391	175.4

a) Percentage change between 2020 and 2021. Source: Mortality Information System (Sistema de Informações sobre Mortalidade - SIM).

## DISCUSSION

In 2020 and 2021, there were excess deaths in the state of Santa Catarina, higher in males and for the variable age, in individuals aged 60 years and older. It could be seen excess deaths and an increasing death ratio for most macro-regions of the state and periods, with the exception of the 2^nd^ quarter of 2020 and December 2021. The largest excess deaths occurred in the same periods and locations where there was a higher number of deaths due to COVID-19.

In a context of low testing and lack of standardization in data notification, a technical note pointed out rates of underreporting of deaths in Santa Catarina during the COVID-19 pandemic near 278%.^16^ The excess deaths found in this study was 27.4% higher than the number of cumulative deaths due to COVID-19 and reported by the state. A possible explanation for this lies in the fact that excess deaths are not an indicator affected by the classification of causes of death, therefore they have not been affected by the high underreporting.^11^ ^,^ ^12^ In addition, excess deaths encompass not only deaths due to COVID-19, but may also be a reflection of indirect mortality, resulting, among other factors, from health system overload that led to an increase in deaths from other conditions.^3^

Studies show the association between higher COVID-19 morbidity and mortality and regions and locations with high population density, where there are usually higher transmission rates and lower social isolation.^17^ ^-^ ^19^ In this study, the macro-regions with the highest demographic density were those with the highest excess deaths and most deaths due to COVID-19, in line with what is described in the literature.^17^ ^-^ ^20^ Great West and Midwest and Serra, although they are locations of low population density, showed high excess deaths, especially in 2021, and possible explanations for this finding would be the intense flow of people and unfavorable sanitary conditions in meat industries in the region, which are favorable to the spread of the virus.^20^ ^-^ ^24^

The first death due to COVID-19 in the state of Santa Catarina occurred in March 2020, when the social isolation rate reached 72.8% - probably attributed to the implementation of social distancing measures.^23^ ^,^ ^25^ ^,^ ^26^ However, a study indicated inconsistency with the reality of the pandemic at the time of publication of state decrees, especially in the 2^nd^ half, with early flexibility and partial restrictions. At the end of the year, there was also government and media encouragement of tourism, increasing the risk of spreading the virus.^27^ This scenario seems to explain the evolution of excess deaths in the state in 2020, with higher surplus in the 2^nd^ half of the year.^27^

In the state of Santa Catarina, the stratification of excess deaths by sex and age showed excess deaths in males and growth with increasing age. It is commonly known that the risk of dying from COVID-19 is higher in males, associated with hormonal, immunological factors and their lower tendency to self-care.^28^ The risk of death is also higher in older adults, given that, in addition to the natural consequences of aging, there is a high prevalence of comorbidities in this age group, making it more susceptible to both COVID-19 and lack of healthcare services during the pandemic.^29^

As a limitation, it is noteworthy that the way to estimate the excess deaths is not among the most robust ones, although it is pointed out as a standard and effective approach.^15^ Secondary databases are also directly influenced by the quality of filling out, and their heterogeneous coverage in the territory. As a potentiality, the analyzed indicator has been pointed out as one of the most important in the COVID-19 pandemic scenario, because, in addition to capturing the direct and indirect impact of mortality due to the pandemic, it is independent of testing strategies or the final classification of causes of death.^11^ ^-^ ^13^

It can be concluded that there were excess deaths in Santa Catarina and in all its macro-regions during the years 2020 and 2021, in most of the periods analyzed, higher in males and in those aged 60 years and older.
