# Erratum

**DOI:** 10.1590/S2237-96222024V33E202479.en

**Published:** 2024-06-10

In the article ***Excess deaths among adults in the state of Santa Catarina: an ecological study during the COVID-19 pandemic, Brazil, 2020-2021*** , doi 10.1590/S2237-96222023000200003, published on Epidemiologia e Serviços de Saúde: revista do SUS, 32(2):e2023360, 2023, on pages 07 to 09, references:

Original text:

1. Ministério da Saúde (BR). Secretaria de Vigilância em Saúde. Departamento de Doenças de Condições Crônicas e Infecções Sexualmente Transmissíveis. Brasil Livre da Tuberculose: Plano Nacional pelo Fim da Tuberculose como Problema de Saúde Pública: Estratégias para 2021-2025. Brasília: Ministério da Saúde; 2021 [citado 2022 Maio 17]. Disponível em: https:// www.gov.br/saude/pt-br/centrais-de-conteudo/publicacoes/publicacoes-svs/tuberculose/ plano-nacional-pelo-fim-da tuberculose-como-problema-de- saude-publica_-estrategias-para-2021-2925.pdf/view

2. Trauer JM, Dodd PJ, Gomes MGM, Gomes GB, Houben RMGJ, McBryde EM, et al. The impor- tance of heterogeneity to the epidemiology of tuberculosis. Clin Infect Dis. 2019;69(1):159-66. doi: 10.1093/cid/ciy938

3. Zhang Q, Song W, Liu S, An Q, Tao N, Zhu X, et al. An ecological study of tuberculosis in- cidence in China, from 2002 to 2018. Front Public Health. 2022;18(9):766362. doi: 10.3389/ fpubh.2021.766362

4. Benchimol EI, Smeeth L, Guttmann A, Harron K, Moher D, Petersen I, et al. The REporting of studies Conducted using Observational Routinely-collected health Data (RECORD) state- ment. PLoS Med. 2015;12(10):e1001885. doi: 10.1371/journal.pmed.1001885

5. Instituto Brasileiro de Geografia e Estatística. Cidades e estados: Paraná. Rio de Janeiro: Instituto Brasileiro de Geografia e Estatística; 2022 [citado 2023 Nov 23]. Disponível em: https:// www.ibge.gov.br/cidades-e-estados/pr.html

6. Santos L. Região de saúde e suas redes de atenção: modelo organizativo-sistêmico do SUS. Cien Saude Colet. 2017;22(4):1281-9. doi: 10.1590/1413-81232017224.26392016

7. Khan MK, Islam MN, Ferdous J, Alam MM. An overview on epidemiology of tuberculosis. Mymensingh Med J. 2019;28(1):259-66.

8. Prado Junior JC, Medronho RA. Spatial analysis of tuberculosis cure in primary care in Rio de Janeiro, Brazil. BMC Public Health. 2021;21(1):1841. doi: 10.1186/s12889-021-11834-1

9. Pereira TV, Nogueira MC, Campos EMS. Spatial analysis of tuberculosis and its relationship with socioeconomic indicators in a medium-sized city in Minas Gerais. Rev Bras Epidemiol. 2021;24(suppl1):e210021. doi: 10.1590/1980-549720210021.supl.1

10. Nogueira VD, Xavier-Gomes LM, Barbosa TLA. Mortalidade por homicídios em linha de fronteira no Paraná, Brasil. Cien Saude Colet. 2020;25(8):3107-18. doi: 10.1590/1413-81232020258.28522018

11. Aikes S, Rizzotto MLF. Integração regional em cidades gêmeas do Paraná, Brasil, no âmbito da saúde. Cad Saude Publica. 2018;34(8):e00182117. doi: 10.1590/0102-311X00182117

12. Hortelan MS, Almeida ML, Fumincelli L, Zilly A, Nihei OK, Peres AM, et al. Papel do gestor de saúde pública em região de fronteira: scoping review. Acta Paul Enferm. 2019;32(2):229-36. doi: 10.1590/1982-0194201900031

13. Gelaw YA, Yu W, Magalhães RJS, Assefa Y, Williams G. Effect of temperature and altitude dif- ference on tuberculosis notification: a systematic review. J Glob Infect Dis. 2019;11(2):63-8. doi: 10.4103/jgid.jgid_95_18

14. Magnabosco GT, Órfão NH, Brunello MEF, Wysocki AD, Lopes LM, Campoy LT. Novas doen- ças e ameaças antigas: a repercussão da covid-19 no manejo da tuberculose. Saude Coletiva (Barueri). 2020;10(54):2639-44. doi: 10.36489/saudecoletiva.2020v10i54p2639-2644

15. Siqueira TC, Martellet MG, Tavernard GLN, Silva VM, Moura STS, Silva LAF, et al. Percepção de enfermeiros: enfoque na família e orientação para a comunidade nas ações de tuberculose. Cienc Cuid Saude.2020;19:e50175. doi: 10.4025/ciencuidsaude.v19i0.50175

16. Silva GDM, Duarte EC, Cruz OG, Garcia LP. Identificação de microrregiões com subnotificação de casos de tuberculose no Brasil, 2012 a 2014. Epidemiol Serv Saude. 2020;29(1):e2018485. doi: 10.5123/S1679- 49742020000100025

17. Arentz M, Ma J, Zheng P, Vos T, Murray CJL, Kyu HH. The impact of the COVID-19 pandemic and associated suppression measures on the burden of tuberculosis in India. BMC Infect Dis. 2022;22(1):92. doi: 10.1186/s12879-022-07078-y

18. Lakoh S, Jiba DF, Baldeh M, Adekanmbi O, Barrie U, Seissay AL, et al. Impact of COVID-19 on tuberculosis case detection and treatment outcomes in Sierra Leone. Trop Med Infect Dis. 2021;6(3):154. doi: 10.3390/tropicalmed6030154

19. Souza ASR, Amorim MMR, Melo ASO, Delgado AM, Forêncio ACMCC, Oliveira TV, et al. Aspectos gerais da pandemia da covid-19. Rev Bras Saude Mater Infant. 2021;21(Suppl 1):529-46. doi: 10.1590/1806-9304202100S100003

20. Couto MT, Barbieri CLA, Matos CCSA. Considerações sobre o impacto da covid-19 na re- lação indivíduo-sociedade: da hesitação vacinal ao clamor por uma vacina. Saude Soc. 2021;30(1):e200450. doi: 10.1590/S0104-12902021200450

21. Jain VK, Iyengar KP, Samy DA, Vaishya R. Tuberculosis in the era of COVID-19 in India. Diabetes Metab Syndr. 2020;14(5):1439-43. doi: 10.1016/j.dsx.2020.07.034

22. Fei H, Yinyin X, Hui C, Ni W, Xin D, Wei C, et al. The impact of the COVID-19 epidemic on tuberculosis control in China. Lancet Reg Health West Pac. 2020;3:100032 doi: 10.1016/j. lanwpc.2020.100032

23. Governo do Estado (SC). Secretaria do Estado de Saúde de Santa Catarina. Coronavírus [Internet]. 2020 [atualizado 2022 Mar 8; citado 2022 Mar 8]. Disponível em: http://www.corona- virus.sc.gov.br/

24. Ripplinger F, Dalmora TWR, Scherma RA. Geografia da covid-19 em Santa Catarina: notas sobre o trabalho na criação e na indústria de abates de animais. Revista Pegada. 2020;21(2):463-92. doi:10.33026/peg.v21i2.7816

25. Inloco. Mapa brasileiro da COVID-19 [Internet]. [s.l.]: Inloco; 2020 [atualizado 2022 Mar 8; citado 2022 Mar 8]. Disponível em: https://mapabrasileirodacovid.inloco.com.br/pt/

26. Hughes HMFBR, Carneiro RAVD, Hillesheim D, Hallal ALC. Evolução da COVID-19 em Santa Catarina: decretos estaduais e indicadores epidemiológicos até agosto de 2020. Epidemiol Serv Saude. 2021;30(4):e2021521. doi: 10.1590/S1679-49742021000400025

27. Caponi S. Covid-19 em Santa Catarina: um triste experimento populacional. Hist Cienc Saude Manguinhos. 2021;28(2):593-8. doi: 10.1590/S0104-59702021005000004

28. Bwire GM. Coronavirus: why men are more vulnerable to covid-19 than women?. SN Comp Clin Med. 2020;2(7):874-6. doi: 10.1007/s42399-020-00341-w

29. Perrotta F, Corbi G, Mazzeo G, Boccia M, Aronne L, D’Agnano V, et al. COVID-19 and the elderly: insights into pathogenesis and clinical decision-making. Aging Clin Exp Res. 2020;32(8):1599- 608. doi: 10.1007/s40520-020-01631-y

Corrected text:

1. Ministério da Saúde (BR). Secretaria de Vigilância em Saúde. Departamento de Vigilância Epidemiológica. Painel Coronavírus [Internet]. Brasília: Ministério da Saúde; 2022 [citado 2022 Mar 8]. Disponível em: https://covid.saude.gov.br/

2. World Health Organization. WHO coronavirus (COVID-191) dashboard [Internet]. Genebra: World Health Organization; 2022 [cited 2022 Mar 8]. Available from: https://covid19.who.int/

3. Hacker KA, Briss PA, Richardson L, Wright J, Petersen R. COVID-19 and chronic disease: the impact now and in the future. Prev Chronic Dis. 2021;18:e62. doi: 10.5888/pcd18.210086

4. Giovanella L, Bousquat A, Medina MG, Mendonça MHM, Facchini LA, Tasca R, et al. Desafios da atenção básica no enfrentamento da pandemia de COVID-19 no SUS. In: Portela MC, Reis LGC, Lima SML, editores. Covid-19: desafios para a organização e repercussões nos sistemas e serviços de saúde [online]. Rio de Janeiro: Observatório Covid-19 Fiocruz, Editora Fiocruz; 2022. p. 201-16. doi: 10.7476/9786557081587.0013

5. Castro R. Observatório Covid-19 aponta maior colapso sanitário e hospita- lar da história do Brasil [Internet]. Rio de Janeiro: Fundação Oswaldo Cruz; 2021 [atualizado 2021 Mar 17, citado 2022 mar 8]. Disponível em: https://portal.fiocruz.br/noticia/ observatorio-covid-19-aponta-maior-colapso-sanitario-e-hospitalar-da-historia-do-brasil

6. Rodrigues JN, Azevedo DA. Pandemia do Coronavírus e (des)coordenação federativa: evi- dências de um conflito político-territorial. Espaço e Economia. 2020;18:1-11. doi: 10.4000/ espacoeconomia.12282

7. Guimarães NS, Carvalho TML, Machado-Pinto J, Lage R, Bernardes RM, Peres, ASS, et al. Aumento de óbitos domiciliares devido a parada cardiorrespiratória em tempos de pandemia de COVID-19. Arq Bras Cardiol. 2021;116(2):266-71. doi: 10.36660/abc.20200547

8. Bispo Júnior JP, Santos DB. COVID-19 como sindemia: modelo teórico e fundamen- tos para a abordagem abrangente em saúde. Cad Saude Publica. 2021;37(10):1-14. doi: 10.1590/0102-311X00119021

9. Mafham MM, Spata E, Goldacre R, Gair D, Curnow P, Bray M, et al. COVID-19 pandemic and admission rates for and management of acute coronary syndromes in England. Lancet. 2020;396(10248):381-9. doi: 10.1016/S0140-6736(20)31356-8

10. Noronha KVMS, Guedes GR, Turra CM, Andrade MV, Botega L, Nogueira D, et al. Pandemia por COVID-19 no Brasil: análise da demanda e da oferta de leitos hospitalares e equipamentos de ventilação assistida segundo diferentes cenários. Cad Saude Publica. 2020;36(6):e00115320. doi: 10.1590/0102-311X00115320

11. Statistical Office of the European Union. Excess Mortality - Statistics. Eurostat - Statistics Explained [Internet]. [Luxemburgo] Statistical Office of the European Union; 2022 jul [ci- ted 2022 July 2]. Available from: https://ec.europa.eu/eurostat/statistics-explained/index. php?title=Excess_mortality_-_statistics

12. Beaney T, Clarke JM, Jain V, Golestaneh AK, Lyons G, Salman D, et al. Excess mortality: the gold standard in measuring the impact of COVID-19 worldwide?.J R Soc Med. 2020;113(9):329-34. doi: 10.1177/0141076820956802

13. World Health Organization. Revealing the Toll of COVID-19: a technical package for rapid mor- tality surveillance and epidemic response [Internet]. Genebra: World Health Organization; 2020 [update 2020 May 21, cited 2022 mar 8]. 30 p. Available from: https://www.who.int/ publications/i/item/revealing-the-toll-of-covid-19

14. Ludvigsson JF. Systematic review of COVID-19 in children shows milder cases and a better prognosis than adults. Acta Paediatr. 2020;109(6):1088-95. doi: 10.1111/apa.15270

15. National Records of Scotland. Choosing a five year average for the measurement of excess deaths [Internet]. [Edinburgh]:National Records of Scotland; 2022 [cited 2022 Apr 13]. Available from: https://www.nrscotland.gov.uk/files/statistics/covid19/covid-deaths-methodology-ex- cess-deaths-in-2022.pdf

16. Nogueira AL, Nogueira CL, Zibetti AW, Roqueiro N, Bruna-Romero O, Carciofi BAM. Estimativa da Subnotificação de Casos da COVID-19 no Estado de Santa Catarina [Internet]. Joinville: Universidade Federal de Santa Catarina; 2020 [atualizado 2020 Jul 23; citado 2023 out 23]. Disponível em: https://covid19sc.github.io/subnotificacao_covid19.html

17. Wong DWS, Li Y. Spreading of COVID-19: density matters. PLoS One. 2020;15(12):e0242398. doi: 10.1371/journal.pone.0242398

18. Chen K, Li Z. The spread rate of SARS-CoV-2 is strongly associated with population density. J Travel Med. 2021;27(8):taaa186. doi: 10.1093/jtm/taaa186

19. Ilardi A, Chieffi S, Iavarone A, Ilardi CR. SARS-CoV-2 in Italy: population density correlates with morbidity and mortality. Jpn J Infect Dis. 2020;74(1):61-4. doi: 10.7883/yoken.JJID.2020.200

20. Serviço Brasileiro de Apoio às Micro e Pequenas Empresas. Santa Catarina em números [Internet]. Florianópolis: Serviço Brasileiro de Apoio às Micro e Pequenas Empresas. 2013 [citado 2022Mar8]. Disponívelem: https://www.sebrae.com.br/sites/PortalSebrae/ufs/sc/quem_somos/ santa-catarina-em-numeros,2fedd49dc3246410VgnVCM2000003c74010aRCRD

21. Moura PH, Luz RA, Gai MJP, Klokner S, Torrico G, KnapiK J, et al. Perfil epidemiológico da co- vid-19 em Santa Catarina. RIES. 2020;9(19):163-80. doi: 10.33362/ries.v9i1.2316

22. Merêncio I, Monteiro GM, Vieira CAO. Aglomerados ativos de COVID-19 em Santa Catarina, Brasil, e tendência de mobilidade dos locais de trabalho. Cad Saude Publica. 2021;37(6):1-13. doi: 10.1590/0102-311X00301620

23. Governo do Estado (SC). Secretaria do Estado de Saúde de Santa Catarina. Coronavírus [Internet]. 2020 [atualizado 2022 Mar 8; citado 2022 Mar 8]. Disponível em: http://www.corona- virus.sc.gov.br/

24. Ripplinger F, Dalmora TWR, Scherma RA. Geografia da covid-19 em Santa Catarina: notas sobre o trabalho na criação e na indústria de abates de animais. Revista Pegada. 2020;21(2):463-92. doi: 10.33026/peg.v21i2.7816

25. Inloco. Mapa brasileiro da COVID-19 [Internet]. [s.l.]: Inloco; 2020 [atualizado 2022 Mar 8; citado 2022 Mar 8]. Disponível em: https://mapabrasileirodacovid.inloco.com.br/pt/

26. Hughes HMFBR, Carneiro RAVD, Hillesheim D, Hallal ALC. Evolução da COVID-19 em Santa Catarina: decretos estaduais e indicadores epidemiológicos até agosto de 2020. Epidemiol Serv Saude. 2021;30(4):e2021521. doi: 10.1590/S1679-49742021000400025

27. Caponi S. Covid-19 em Santa Catarina: um triste experimento populacional. Hist Cienc Saude Manguinhos. 2021;28(2):593-8. doi: 10.1590/S0104-59702021005000004

28. Bwire GM. Coronavirus: why men are more vulnerable to covid-19 than women?. SN Comp Clin Med. 2020;2(7):874-6. doi: 10.1007/s42399-020-00341-w

29. Perrotta F, Corbi G, Mazzeo G, Boccia M, Aronne L, D’Agnano V, et al. COVID-19 and the elderly: insights into pathogenesis and clinical decision-making. Aging Clin Exp Res. 2020;32(8):1599- 608. doi: 10.1007/s40520-020-01631-y

